# Pure and Reliable Medicines

**Published:** 1881-01

**Authors:** 


Pure and Reliable Medicines.—Messrs. Sharp & Dohme of this city, have favored us with samples of their sugar-coated pills and granules; and also specimens of their fluid and solid extracts, for which we return thanks. We have been accustomed to prescribe and to recommend their preparations to our patients for several years past, as we know them to be reliable.

